# Screening a Panel of Topical Ophthalmic Medications against MMP-2 and MMP-9 to Investigate Their Potential in Keratoconus Management

**DOI:** 10.3390/molecules27113584

**Published:** 2022-06-02

**Authors:** Amany Belal, Mohamed A. Elanany, Eman Y. Santali, Ahmed A. Al-Karmalawy, Moustafa O. Aboelez, Ali H. Amin, Magda H. Abdellattif, Ahmed B. M. Mehany, Hazem Elkady

**Affiliations:** 1 Department of Pharmaceutical Chemistry, College of Pharmacy, Taif University, P.O. Box 11099, Taif 21944, Saudi Arabia; eysantali@tu.edu.sa; 2 School of Pharmacy and Pharmaceutical Industries, Badr University in Cairo (BUC), Cairo 11884, Egypt; mohamed.a.elanany@hotmail.com; 3 Department of Pharmaceutical Medicinal Chemistry, Faculty of Pharmacy, Horus University-Egypt, New Damietta 34518, Egypt; akarmalawy@horus.edu.eg; 4 Department of Pharmaceutical Chemistry, Faculty of Pharmacy, Sohag University, Sohag 82524, Egypt; moustafaaboelez@pharm.sohag.edu.eg; 5 Deanship of Scientific Research, Umm Al-Qura University, Makkah 21955, Saudi Arabia; ahamin@uqu.edu.sa; 6 Zoology Department, Faculty of Science, Mansoura University, Mansoura 35516, Egypt; 7 Department of Chemistry, College of Sciences, Taif University, P.O. Box 11099, Taif 21944, Saudi Arabia; m.hasan@tu.edu.sa; 8 Zoology Department, Faculty of Science (Boys), Al-Azhar University, Cairo 11884, Egypt; abelal_81@azhar.edu.eg; 9 Pharmaceutical Medicinal Chemistry & Drug Design Department, Faculty of Pharmacy (Boys), Al-Azhar University, Cairo 11884, Egypt; hazemelkady@azhar.edu.eg

**Keywords:** keratoconus, MMP-2, MMP-9, molecular docking, molecular dynamics, MM-GBSA calculations, pharmacophore mapping

## Abstract

Keratoconus (KC) is a serious disease that can affect people of any race or nationality, although the exact etiology and pathogenic mechanism are still unknown. In this study, thirty-two FDA-approved ophthalmic drugs were exposed to virtual screening using docking studies against both the MMP-2 and MMP-9 proteins to find the most promising inhibitors as a proposed computational mechanism to treat keratoconus. Matrix metalloproteinases (MMPs) are zinc-dependent proteases, and MMP inhibitors (MMPIs) are usually designed to interact with zinc ion in the catalytic (CAT) domain, thus interfering with enzymatic activity. In our research work, the FDA-approved ophthalmic medications will be investigated as MMPIs, to explore if they can be repurposed for KC treatment. The obtained findings of the docking study suggest that atenolol and ampicillin are able to accommodate into the active sites of MMP-2 and MMP-9. Additionally, both exhibited binding modes similar to inhibitors used as references, with an ability to bind to the zinc of the CAT. Molecular dynamic simulations and the MM-GBSA binding free-energy calculations revealed their stable binding over the course of 50 ns. An additional pharmacophoric study was carried out on MMP-9 (PDB ID: 1GKC) using the co-crystallized ligand as a reference for the future design and screening of the MMP-9 inhibitors. These promising results open the door to further biological research to confirm such theoretical results.

## 1. Introduction

Keratoconus (KC) is the most common primary cause of corneal ectasia. It commonly strikes in one’s second decade of life, affecting people of all races and nationalities. In the general population, the prevalence is estimated to be 54 per 100,000 [1]. Although the exact etiology and pathogenic mechanism are unknown, environmental and genetic variables are considered to play a role in the disease’s progression [2]. Hay fever and allergies are linked to an increased risk, whereas diabetes is thought to be protective [3]. Keratoconus is caused by a combination of genes, with a relatively high prevalence of positive family history. Even though both genders are affected, men appear to be more frequently involved [4].

The disease’s stage and progression determine the treatment of keratoconus. Spectacles can give an acceptable vision to people in the early stages of their condition, and they are beneficial for those with a visual acuity of 20/40 or greater. On the other hand, spectacles cannot rectify irregular astigmatism, and in such circumstances, hard contact lenses can improve the patient’s vision [4,5]. Penetrating or deep anterior lamellar keratoplasty has been the cornerstone of treatment for advanced KC [6]. Collagen cross-linking (CXL) is a new technology recently introduced. Compared to those that were not treated, eyes treated with CXL were less likely to have difficulties with bulging progression [7].

Many investigations have found higher levels of collagenolytic and gelatinolytic activities in laboratory cultures of KC. Collagenases and gelatinases are members of the matrix metalloproteinases (MMPs) family of zinc-dependent proteins [8]. Compared to tears from controls, tears from persons with keratoconus had 1.9 times greater levels of proteolytic activity and overexpression of various MMPs and cytokines [9].

**Figure 1:**
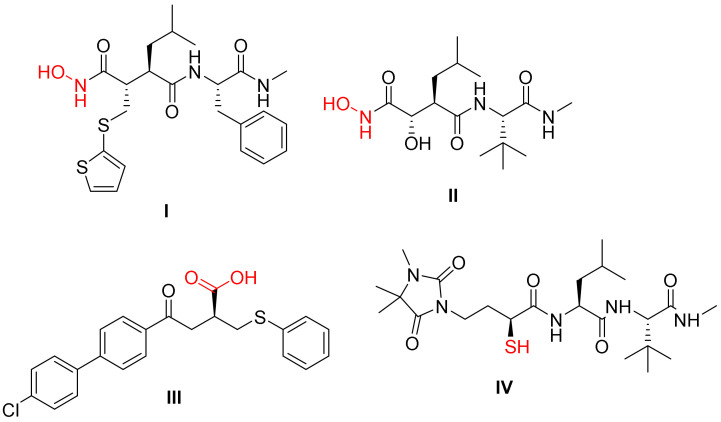
Reported molecules having a group that can interact with zinc in MMPs.

Selective MMP inhibition is an essential objective in medicinal chemistry research [10]. MMP-2 and MMP-9 play important roles in cancer, heart disease, and inflammatory etiology. Many orally accessible broad-spectrum MMP inhibitors (MMPIs) have been discovered in recent years. MMPs typically consist of a pro-peptide sequence, a catalytic (CAT) domain, a hinge region or linker peptide, and a hemopexin domain. MMPs have two zinc ions, one structural zinc and the other in the CAT domain. The early design of the MMPIs relied on the ability of compounds to mimic the amide nature of collagen in addition to having a group that can interact with zinc [11]. In most cases, the hydroxamate group was used for this purpose as in batimastat **I** and marimastat **II** [12]. Other groups were also used for that purpose, such as the carboxylic group of tanomastat **III** and the mercapto group of rebimastat **IV** (Figure 1) [11]. Furthermore, questions have been raised about the real therapeutic efficacy of this family of MMP inhibitors and their considerable toxicity [13]. As a result, researchers are paying more attention to finding novel zinc binding groups (ZBGs) that could be a viable replacement to the hydroxamate function [14,15,16,17,18,19,20].

**Figure 2:**
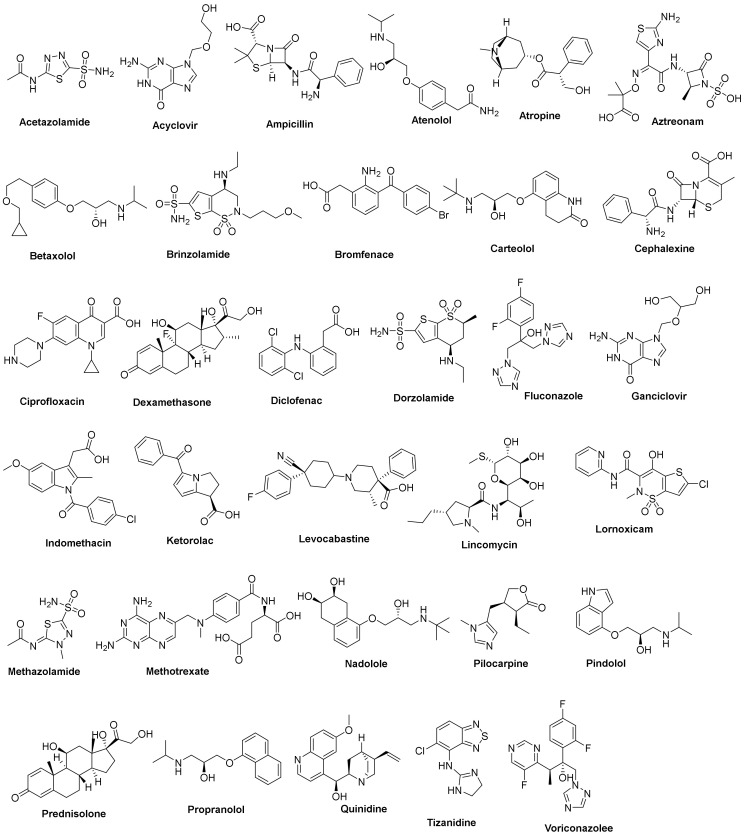
The 2D chemical structures of the 32 FDA-approved drugs used in our in silico study.

The primary strategy of our design implemented the repurposing of various FDA-approved ophthalmic medications for targeting MMP-2 and MMP-9. The first criterium is the market availability of these drugs as ophthalmic systems, which enabled us to shift our whole focus on the pharmacodynamic potentials rather than pharmacokinetics and drug delivery factors. The second criterium is the presence of groups with a high potential of interaction with zinc, such as carboxylic, mercapto, and hydroxyl groups. For these reasons, a group of thirty-two FDA-approved drugs were chosen (Figure 2) [21,22,23]. The selection involved a variety of drugs for different conditions, such as glaucoma (acetazolamide), antivirals (acyclovir and ganciclovir), antibacterials (ampicillin and aztreonam), and analgesics (diclofenac and ketorolac). The drugs were subjected to virtual screening using docking studies against both MMP-2 and MMP-9 to reach a promising candidate against these proteins. The results indicate that some drugs may have potential activities against these proteins, opening the field to further biological studies.

## 2. Results and Discussion

### 2.1. Docking Studies

**Figure 3:**
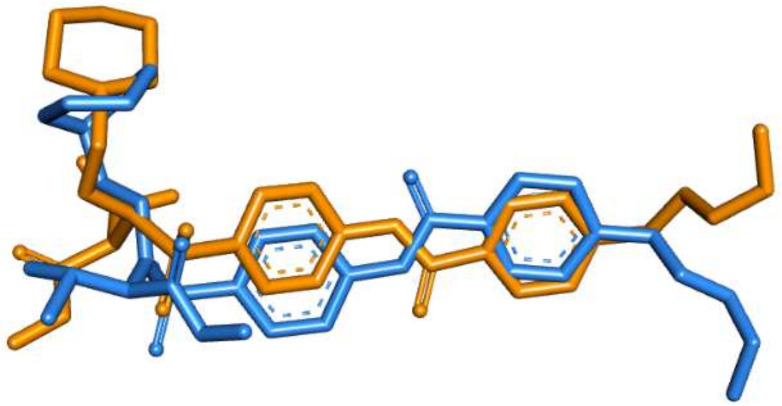
Overlay of the co-crystallized pose (brown) and the re-docked pose (blue) of **I52** inside MMP-2 (PDB ID: 1HOV) during validation (RMSD = 1.30 A^0^).

**Figure 4:**
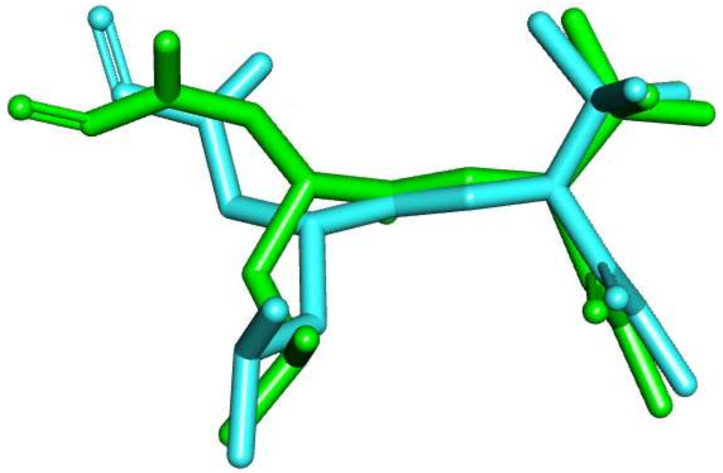
Overlay of the co-crystallized pose (turquoise) and the re-docked pose (green) of **NFH** inside MMP-9 (PDB ID: 1GKC) during validation (RMSD = 0.75 A^0^).

A molecular operating environment (MOE) program was used in the current docking study. The validation of the docking accurately reproduced the binding conformation of the co-crystallized ligands with MMP proteins. The RMSD values were calculated between the co-crystallized poses and the docked poses of the same ligands in MMP-2 (PDB ID: 1HOV) and MMP-9 (PDB ID: 1GKC). Minor deviations of 1.30 and 0.75 A^0^, for MMP-2 and MMP-9, were observed, respectively (Figure 3 and Figure 4). Such results indicated the validity of the docking studies.

#### 2.1.1. Docking of the Target Compounds into MMP-2 Catalytic Domain

All the selected compounds showed favorable binding, demonstrating ∆G (binding free energies) values in negative Kcal.mol^−1^, as shown in Table 1. The most promising candidates were found to be lincomycin, atenolol, and ciprofloxacin, which were able to accommodate into the MMP-2 active site with the highest binding energy score (∆G = −29.06, −28.20, and −27.87 kcal/mol, respectively) and bind the active site essential residues via several hydrogen bonding, electrostatic, and hydrophobic interactions. An analysis of the binding modes of the co-crystalized ligand (**I52**) and our top hits was then performed for a comparative study of how well our compounds conform to the intended design.

**Figure 5:**
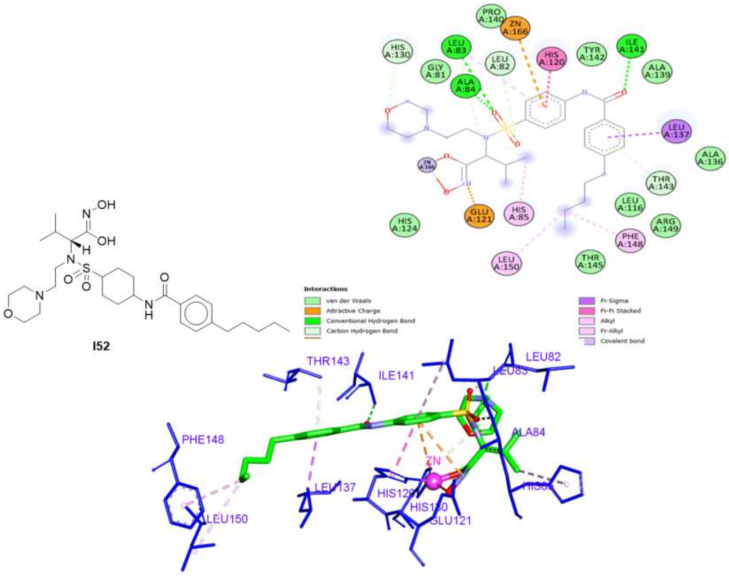
The 2D and 3D interactions of the co-crystallized ligand (**I52**) with amino acid residues of the catalytic domain of MMP-2 (PDB ID: 1HOV).

The 2D and 3D interactions of **I52** (Figure 5) revealed that the pentylbenzamide moiety formed three hydrophobic interactions with Leu137, Phe148, and Leu150, besides two hydrogen bonds with Ile141 and Thr143. Moreover, the sulfamoylphenyl moiety formed three hydrogen bonds with Leu 82, Leu83, and Ala84 and three hydrophobic interactions with Leu83 and His120, and the zinc ion. The morpholine ring interacted with His130 via a hydrogen bond and the isopropyl moiety showed a hydrophobic interaction with His85. Finally, the ZBG (hydroxamate) interacted with the zinc group as well as Glu121 via a hydrophobic interaction.

**Figure 6:**
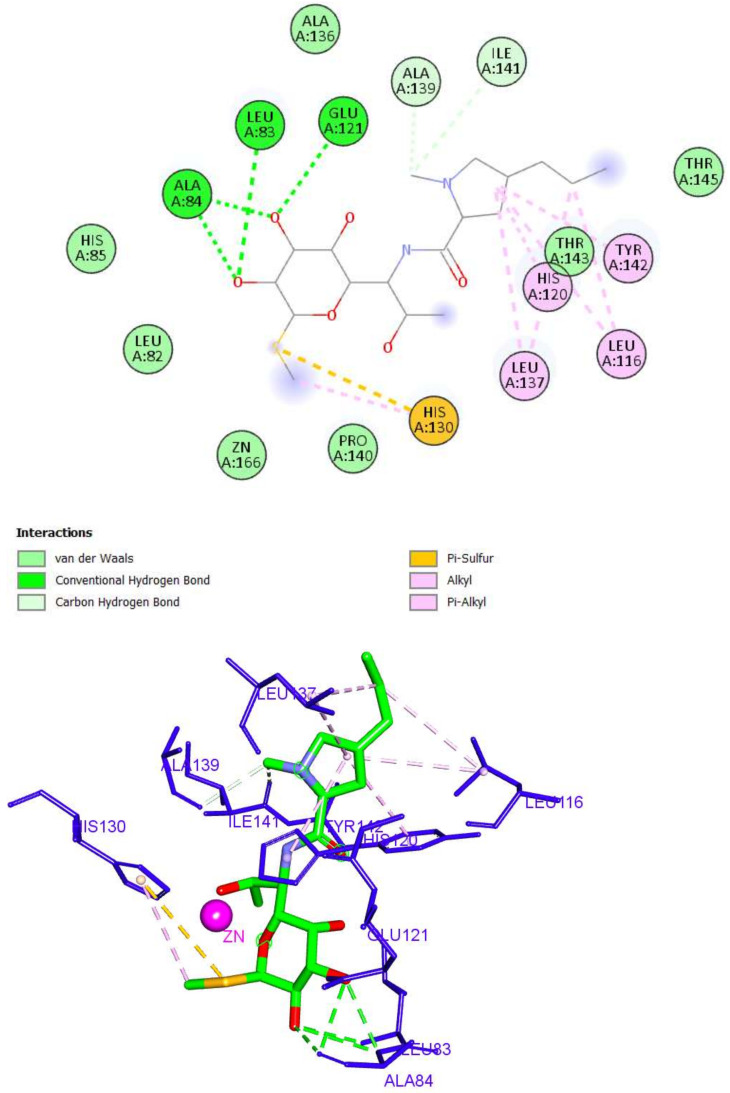
The 2D and 3D interactions of lincomycin with amino acid residues in the catalytic domain of MMP-2 (PDB ID: 1HOV) (hydrogen bonds = green dashed lines, electrostatic interactions = orange dashed lines, pi-pi interactions = deep pink dashed lines, and pi-alkyl interactions = light pink dashed lines).

As described in Figure 6**,** the docking pose of Lincomycin into the MMP-2 active site showed that the *2H*-pyran arm was engaged in six hydrophobic interactions with Leu116, His120, Leu137, and Tyr142, in addition to two hydrogen bonds with Ala139 and Ile141. The sugar moiety formed three hydrogen bonds with Leu83, Ala84, and Glu121. Finally, the methyl thio group formed two hydrophobic bonds with His130. Although lincomycin showed the highest binding score, it lacked the ability to interact with the zinc of the CAT. As a result, it was excluded from subsequent studies.

**Figure 7:**
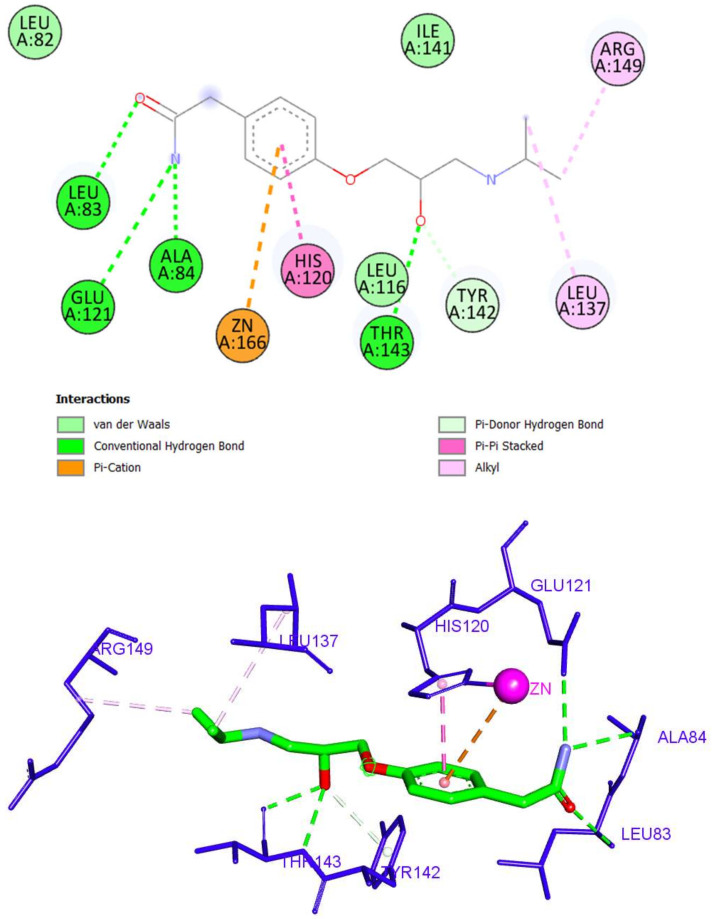
The 2D and 3D interactions of atenolol with amino acid residues in the catalytic domain of MMP-2 (PDB ID: 1HOV).

Although atenolol interacted with the active site with a relatively lower binding, it did also bind successfully to zinc through a hydrophobic pi-cation interaction (Figure 7). Additionally, it formed five hydrogen bonds with Leu83, Ala84, Glu121, Thr142, and Thr143 through its acetamide and hydroxy moieties. Three hydrophobic interactions were observed with His120, Leu137, and Arg149.

**Figure 8:**
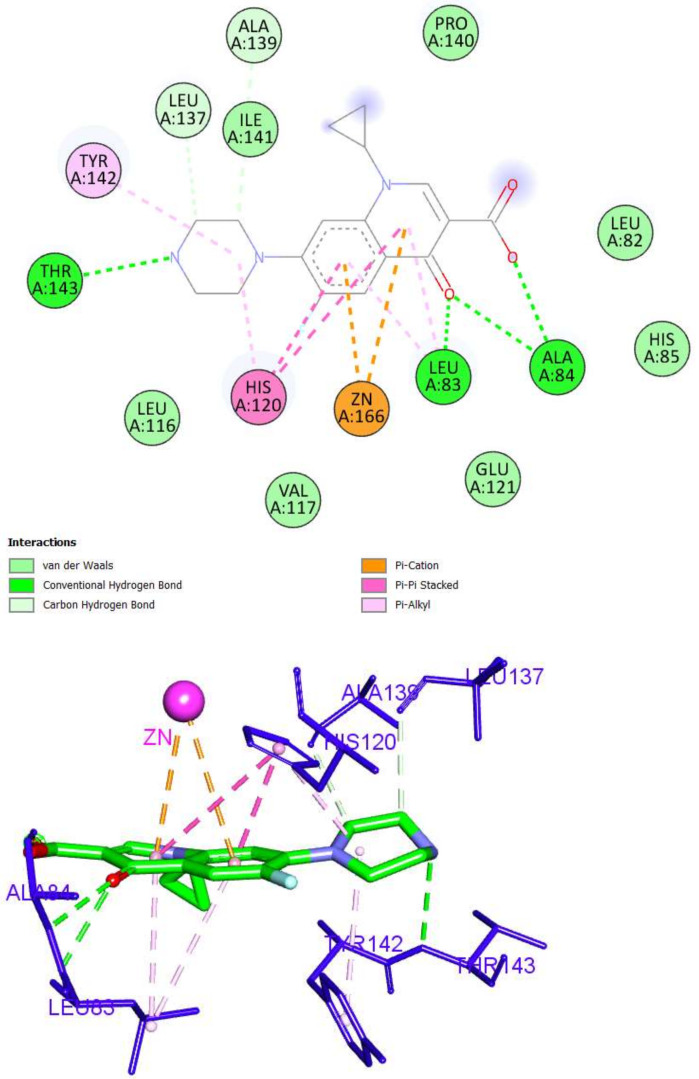
The 2D and 3D interactions of ciprofloxacin with amino acid residues in the catalytic domain of MMP-2 (PDB ID: 1HOV).

Ciprofloxacin (a fluoroquinolone drug) occupied the MMP-2 active site via the formation of six hydrogen bonds and eight hydrophobic interactions. The fluoroquinolone nucleus formed three hydrogen bonds with Leu83 and Ala84, as well as six hydrophobic interactions with zinc, Leu83, and His120. The piperazine arm was incorporated in three hydrogen bonds with Leu137, Ala139, and Thr143, as presented in Figure 8.

#### 2.1.2. Docking of the Target Compounds into MMP-9 Catalytic Domain

The tested compounds revealed to be able to bind into MMP-9 and showed negative ∆G (Kcal.mol^−1^) scores, as shown in Table 2. It was found that ampicillin, aztreonam, and ganciclovir, the most promising candidates, achieved the highest energy score and accommodated into the MMP-9 active site (∆G = −30.81, −29.97, and −28.89 kcal/mol, respectively).

**Figure 9:**
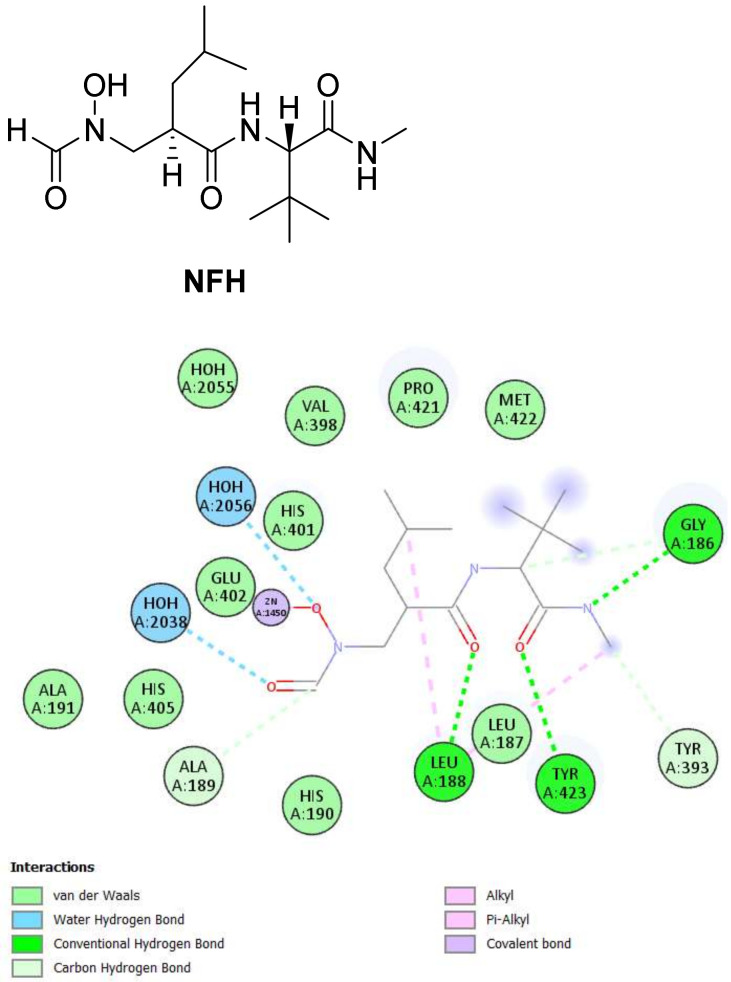

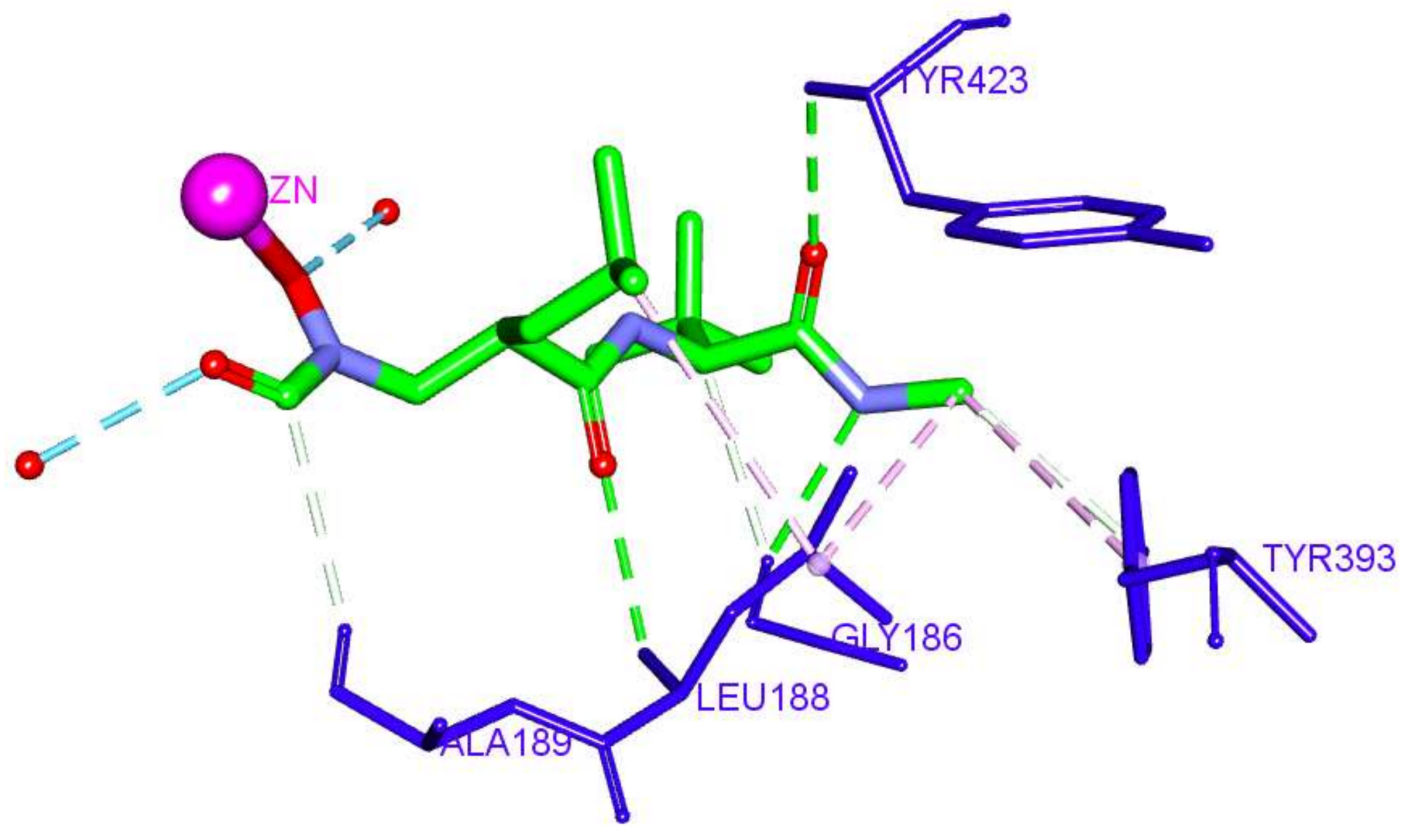
The 2D and 3D interactions of the co-crystallized ligand (**NFH**) with amino acid residues of the catalytic domain of MMP-9 (PDB ID: 1GKC).

By examining the binding interactions of the co-crystalized ligand (**NFH**) to the active site of MMP-9, it showed six hydrogen bonds with Gly186, Leu188, Ala189, Tyr393, and Tyr423, in addition to two hydrophobic interactions with Leu188. The hydroxamate group interacted as expected with the zinc of the CAT, as presented in Figure 9.

**Figure 10:**
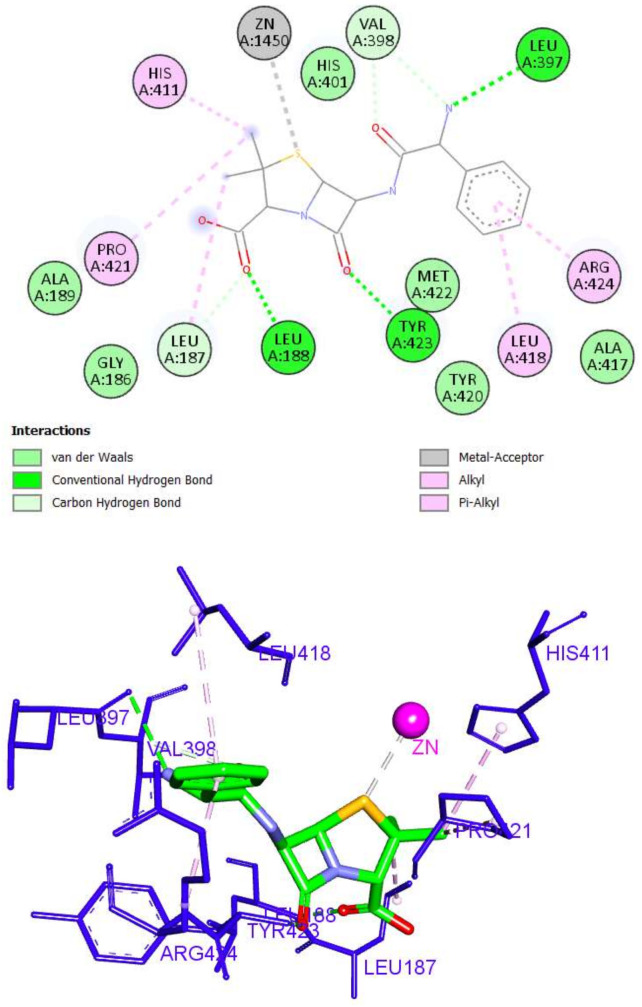
The 2D and 3D interactions of ampicillin with amino acid residues of the catalytic domain of MMP-9 (PDB ID: 1GKC).

This high binding score of ampicillin is attributed to the formation of six hydrogen bonds, five hydrophobic interactions, and zinc binding as well. The 3,3-dimethyl moiety of ampicillin was involved in three hydrophobic bonds with Leu187, His411, and Pro421, while the carboxylic acid and 7-oxo groups formed hydrogen bonds with Leu187, Leu188, and Tyr423. The phenyl acetamide moiety interacted with the active site by three hydrogen bonds with Leu397 and Val398, as well as two hydrophobic interactions with Leu418 and Arg424. Finally, the sulfur atom of the thiazolidine ring was able to interact with the zinc ion of the CAT through a metal–acceptor bond (Figure 10).

**Figure 11:**
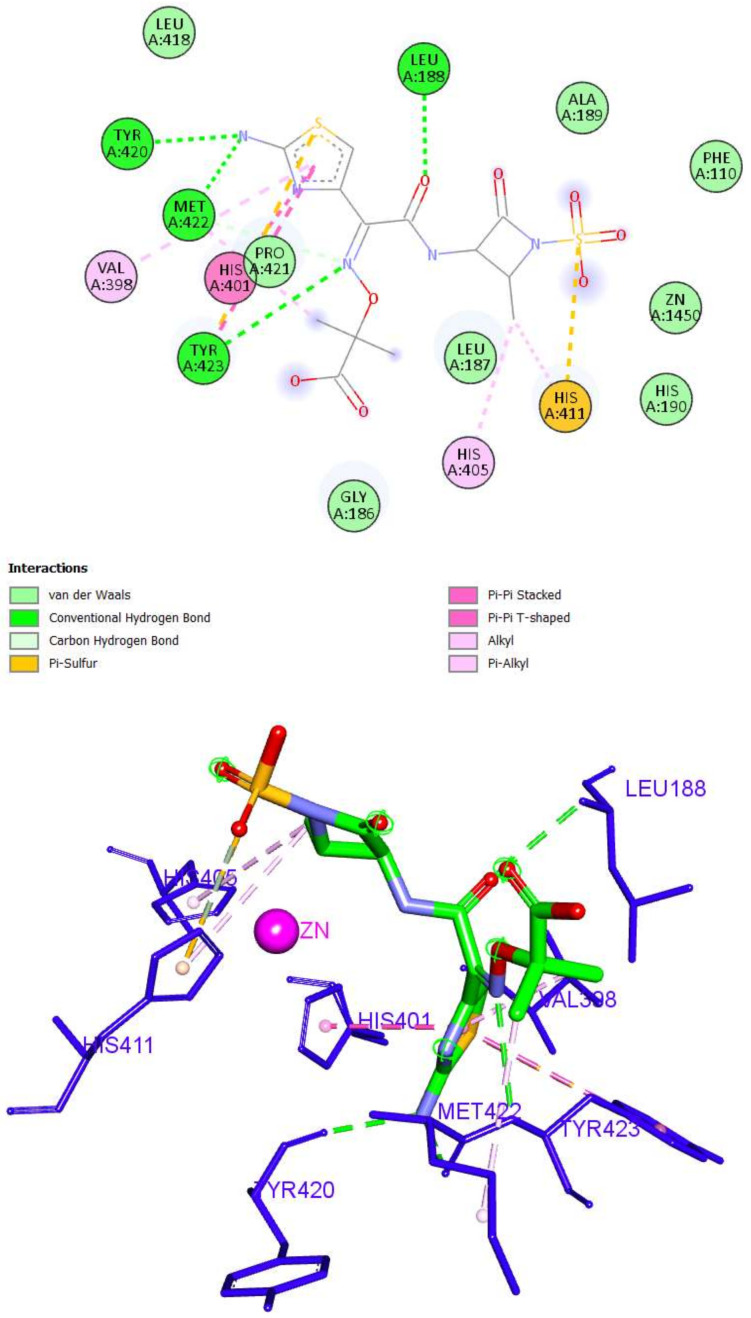
The 2D and 3D interactions of Aztreonam with amino acid residues of the catalytic domain of MMP-9 (PDB ID: 1GKC).

On the other hand, aztreonam was unable to bind to the zinc ion of the CAT. An investigation of the top docking pose of aztreonam showed that it interacted with the MMP-9 active site by forming five hydrogen bond interactions (Leu188, Tyr420, Met422, and Tyr423) and nine hydrophobic interactions (Val398, His401, His405, His411, Met422, and Tyr423) (Figure 11).

**Figure 12:**
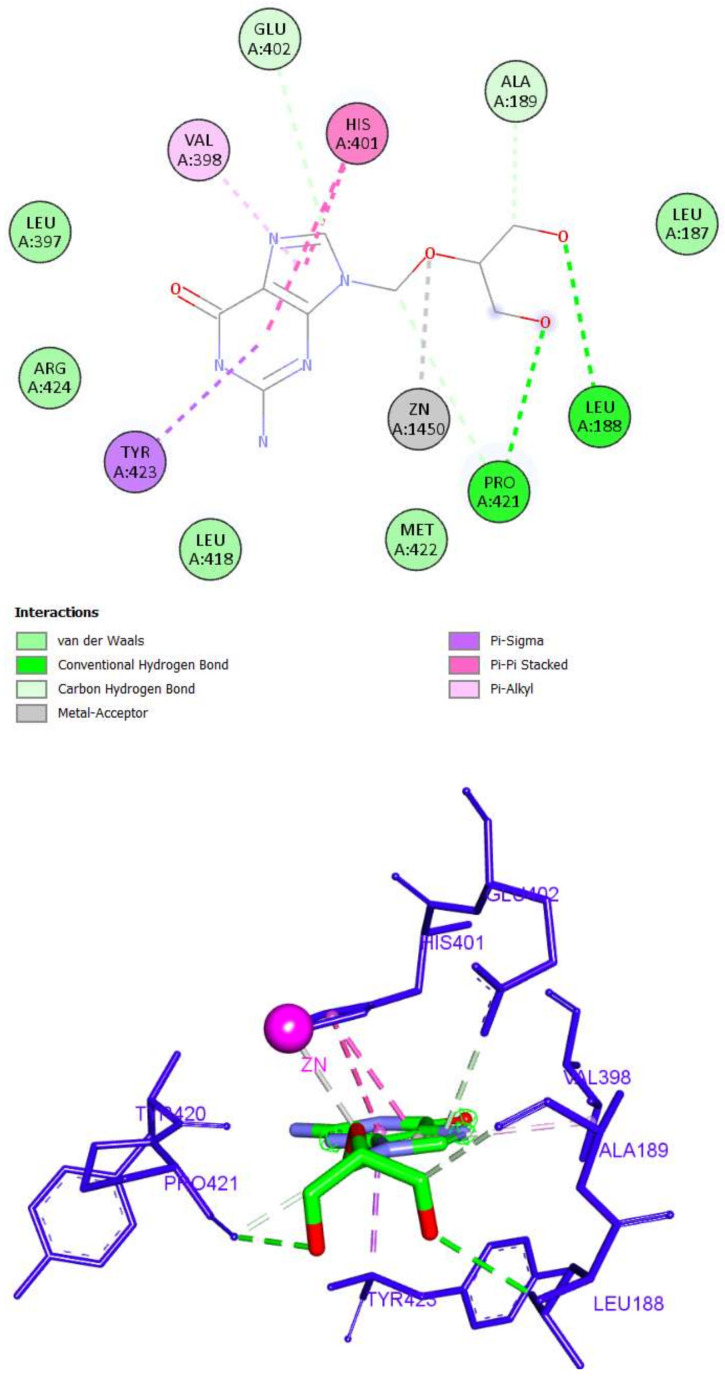
The 2D and 3D interactions of Ganciclovir with amino acid residues of the catalytic domain of MMP-9 (PDB ID: 1GKC).

Finally, the binding of ganciclovir was through four hydrogen bonds with Leu188, Glu402, and Pro421. Four hydrophobic interactions were also detected (Val398, His401, and Tyr423). It was also able to bind with zinc ion through a metal–acceptor interaction (Figure 12).

Because atenolol and ampicillin were the most promising compounds that achieved high docking scores and similar binding modes to co-crystallized ligands with the ability to interact with zinc ions in both enzymes, they were both promoted for further analysis through molecular dynamics.

### 2.2. Molecular Dynamics and Molecular Mechanics–Generalized Born Surface Area (MM-GBSA) Calculations

**Figure 13:**
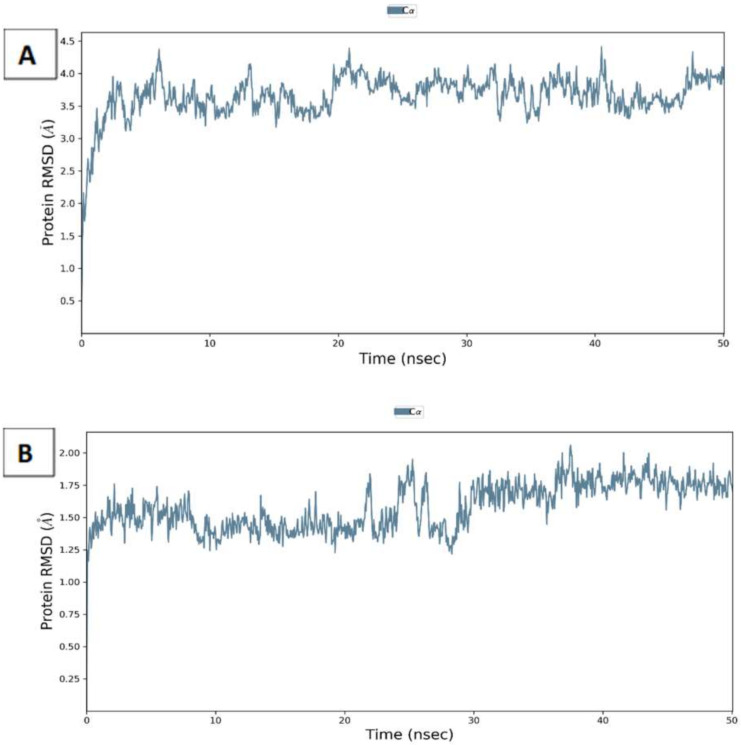
Root mean square deviation (RMSD) of *C*-alpha of MMP-2 (**A**) and MMP-9 (**B**) complexes with atenolol and ampicillin through 50 ns simulations.

The potential of atenolol and ampicillin to bind to MMP-2 and MMP-9, respectively, was further investigated through molecular dynamics. This allows the extensive analysis of the binding modes under realistic physiological conditions. Both protein files with the corresponding drugs were processed by the Schrodinger Maestro suite and simulated for 50 ns. The RMSD of the protein residues (Figure 13) in both complexes showed uniform values around 3.7 and 1.75 A^0^ deviation for MMP-2 and MMP-9, respectively. Furthermore, both atenolol and ampicillin exhibited stable conformations with an RMSD (Supplementary Figures S1 and S2) of around 1.2 and 1.8 A^0^, respectively.

**Figure 14:**
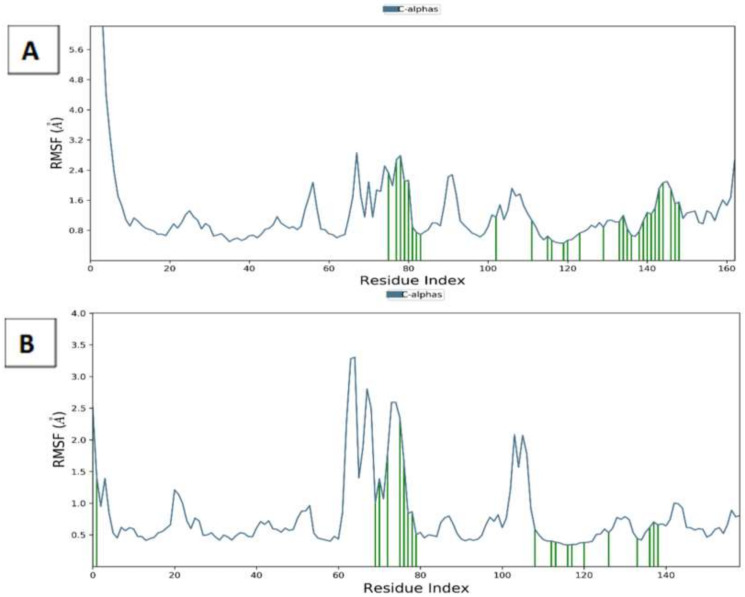
Root mean square fluctuation (RMSF) of *C*-alpha of MMP-2 (**A**) and MMP-9 (**B**) complexes with atenolol and ampicillin through 50 ns simulations. (Ligand contacts are marked green.).

Additionally, the flexibility of the conformers was assessed through the calculation of the root mean square fluctuations (RMSF) of the residues of the proteins and ligand atoms across the simulation time. Consistent with the calculated RMSD, the protein residues showed a low degree of fluctuations, especially with the ones in contact with the ligands as shown in Figure 14. The RMSF of the protein residues showed around 3.5 A^0^ fluctuations of the residues exposed to drugs, while the fluctuations were lower in the case of both ligands, especially ampicillin which showed around 1A^0^ only (Supplementary Figures S3 and S4). These uniform values obtained points to the relative stability of both proteins and drugs conformations for the entire simulation duration.

**Figure 15:**
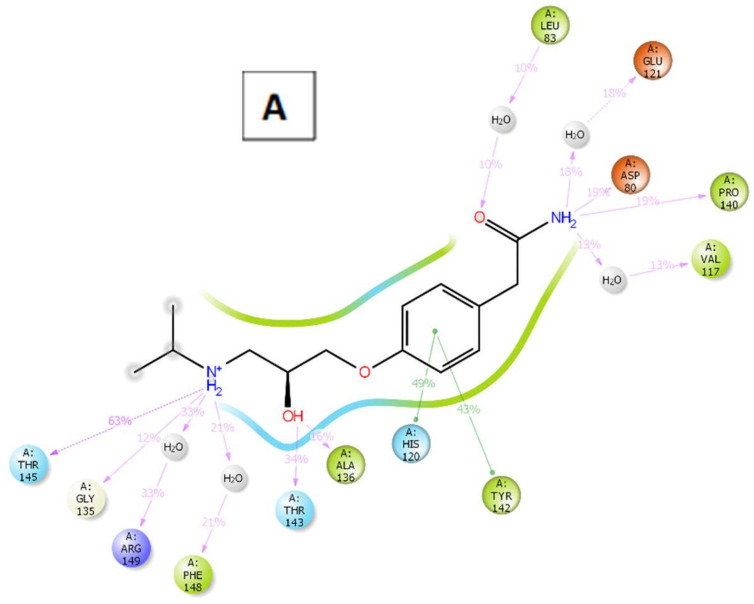

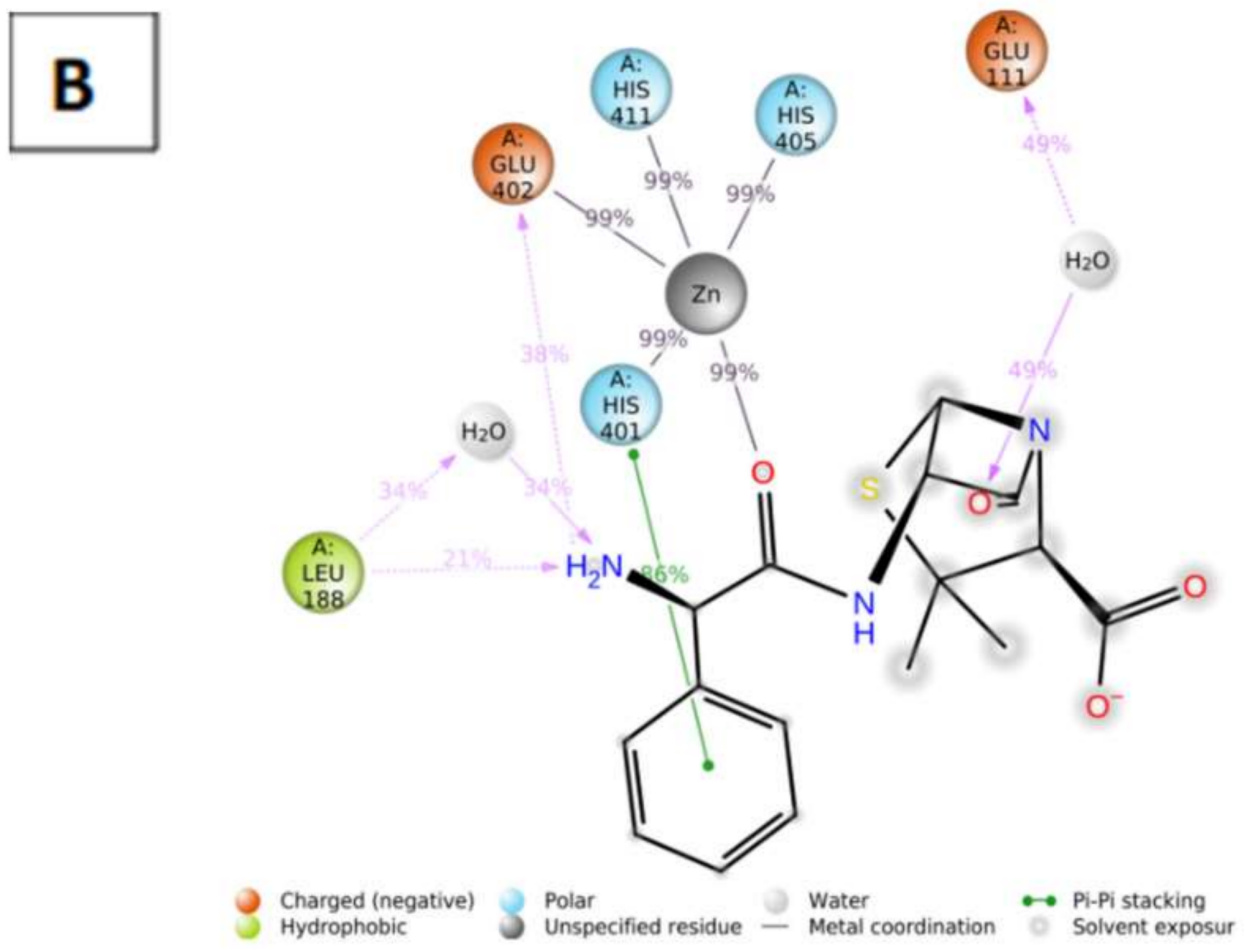
The 2D ligand-protein contact summary of atenolol-MMP-2 (**A**) and ampicillin-MMP-9 (**B**) complexes through 50 ns simulations.

**Figure 16:**
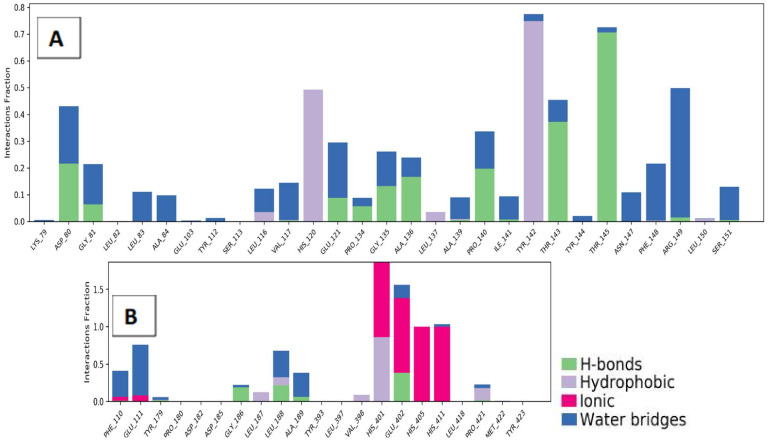
Ligand-protein contact histogram of atenolol-MMP-2 (**A**) and ampicillin-MMP-9 (**B**) complexes through 50 ns simulations.

For a better understanding of the binding modes, a further analysis of the interactions throughout the whole 50 ns simulation time was performed (Figure 15). For MMP-9, ampicillin successfully formed metal coordination with the zinc ion constantly through its amide carbonyl group. On the other hand, the interaction of atenolol with the zinc ion of MMP-2 was not observed; however, it interacted extensively with the binding site residues as well compensating this inability as shown in Figure 16.

One of the most commonly used methods for calculating the binding free energy is molecular mechanics–generalized born surface area (MM-GBSA). The lower a ligand-protein complex’s projected binding free energy is, the more stable the complex is expected to be, and the higher the ligand’s activity and potency (Table 3). Both complexes showed stable binding throughout the dynamic simulation.

### 2.3. Pharmacophore Study

The prospect of repositioning ampicillin for KC treatment may seem beneficial especially for complicated cases suffering from secondary bacterial infections. However, its unattended use for prolonged periods of time increases the risk of bacterial resistance. For this purpose, we extended our study on the MMP-9 inhibitors to propose pharmacophoric features with a high potential of a MMP-9 inhibitory goal for future use.

The co-crystallized ligand of MMP-9 (PDB ID: 1GKC) was used to generate the pharmacophore model using the Discovery studio software. In this test, the protocol of receptor-ligand pharmacophore generation was applied. In this protocol, the software identifies the essential features of **NFH** (co-crystalized ligand) during its interaction with the receptor. The library was then screened, and fit value data were calculated (Supplementary Table S1). Ampicillin, aztreonam, cephalexin, and lincomycin achieved the highest fit value, even comparable to **NFH,** as shown in Table 4.

**Figure 17:**
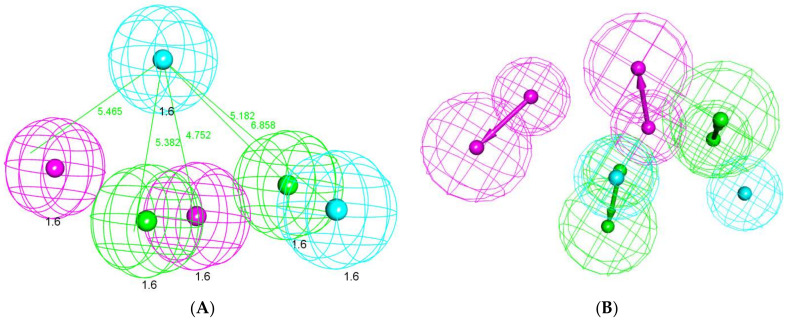
(**A**) The generated 3D pharmacophore geometry with six features: two hydrogen bond donors (pink color) and two hydrogen bond acceptors (green), and two hydrophobic centers (blue). (**B**) The 3D-pharmacophore with vector direction of each feature.

**Figure 18:**
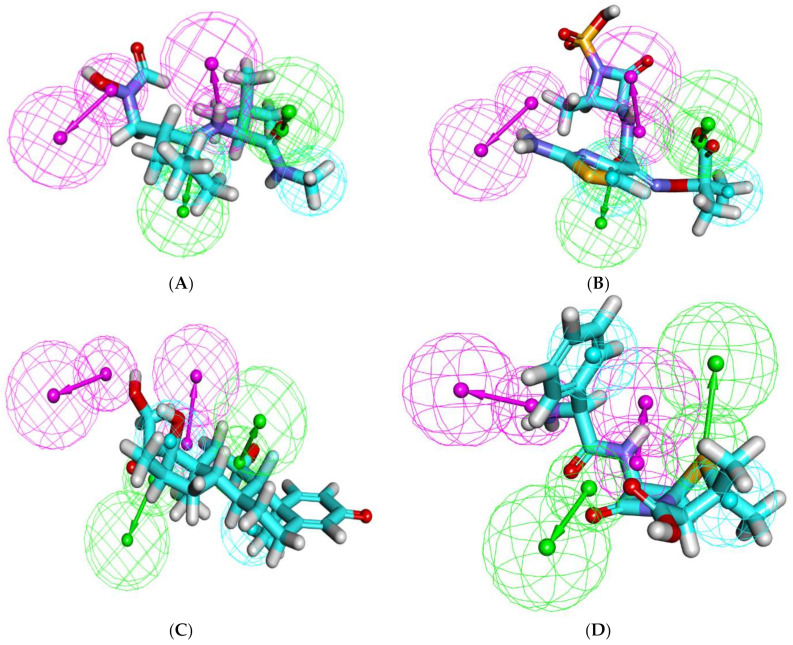

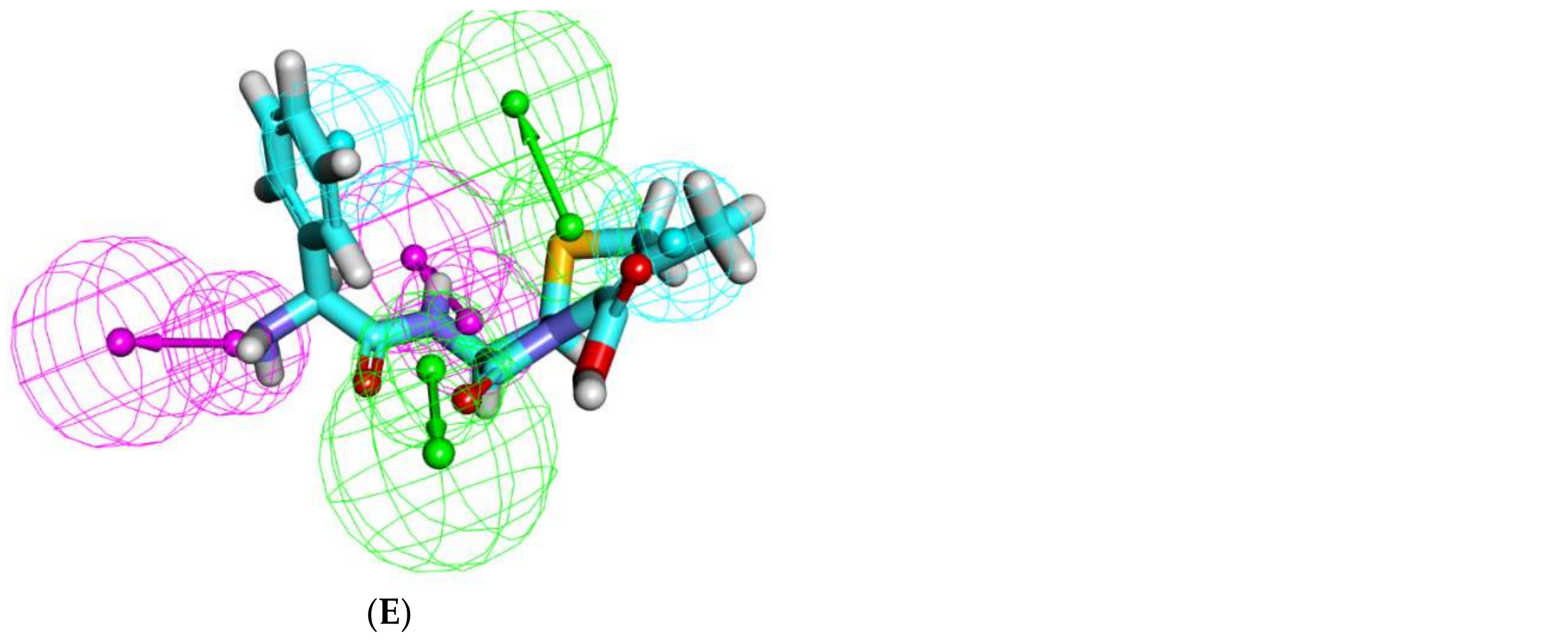
Mapping of the tested compounds on the generated pharmacophore: (**A**) **NFH** on the generated pharmacophore (fit value = 4.74), (**B**) ampicillin (fit value = 3.76), (**C**) aztreonam (fit value = 4.13), (**D**) cephalexin (fit value = 4.06), and (**E**) lincomycin (Fit value = 3.76).

The formed pharmacophore model consisted of six features: two H-bond donors (HBD), two H-bond acceptors (HBA), and two hydrophobic centers (H). The features were distributed in a pyramidal shape with each feature occupying a 1.6 A^0^ radius (Figure 17). In accordance with the data acquired so far, the overlay of each compound on the pharmacophore (Figure 18) demonstrates the ability of the compounds to span across the entire pharmacophoric features in a similar fashion to the co-crystallized inhibitor **NFH**.

## 3. Conclusions

In an attempt to fasten the drug discovery process, a repositioning approach was enforced to discover potential medications for KC in a cost/efficient manner. Because high levels of collagenolytic and gelatinolytic actions were observed, collagenase and gelatinases became a promising target for tackling KC. Among those proteolytic enzymes are MMP-2 and MMP-9. Both are metalloenzymes that are characterized by the presence of zinc ion in the CAT. Thus, inhibition techniques focused on drugs that can interact with zinc ions through their ZBGs. As a result, thirty-two FDA-approved drugs were subjected to virtual screening through docking against MMP-2 and MMP-9 proteins to identify the most promising inhibitors as a proposed computational mechanism to treat KC. The docking results showed the ability of atenolol and ampicillin to accommodate well into the active sites of MMP-2 and MMP-9, respectively. Additionally, both exhibited similar binding modes as **I52** and **NHF** (co-crystallized inhibitors of MMP-2 and MMP-9, respectively) and interacted with the zinc ion of the CAT successfully. Subsequent molecular dynamic simulations and MM-GBSA calculations point to the stability of the binding of both drugs to the respective enzyme, thus adding to the potential of both compounds in KC management. The dual potential properties of ampicillin for the treatment of KC, especially with bacterial infections, pushed for the design of alternatives that could be used for prolonged treatment times without risk of bacterial resistance. An additional pharmacophore study was carried out using the co-crystallized ligand of MMP-9 (PDB ID: 1GKC) as a reference molecule for future designs. These encouraging findings pave the way for additional clinical investigations to confirm such theoretical findings.

## 4. Experimental

### 4.1. Literature Search and Library Generation

The designed compounds’ structures were retrieved online (Pubchem; https://pubchem.ncbi.nlm.nih.gov/) (accessed on 1 April 2022) and sketched using ChemBioDraw Ultra 14.0 and saved in MDL-SD file format.

### 4.2. Docking Studies

The crystal structures of MMP-2 and MMP-9 (PDB ID: 1HOV [16] and PDB ID: 1GKC [17], respectively) were downloaded from the Protein Data Bank (http://www.rcsb.org/pdb) (accessed on 1 April 2022). Molecular operating environment was used for docking. At first, the protein files were prepped using built-in “Quickprep” function. Initial validation was performed through docking of each co-crystallized ligand to its protein file, followed by the calculation of root mean square deviation (RMSD) between the docked pose and the co-crystallized one. After successful validation, the library of compounds was imported and prepped into MOE database file, that was then docked using “Induced fit” protocol. The interactions were then viewed using Discovery Studio Visualizer 2021 [24,25,26,27,28,29,30,31,32].

### 4.3. Molecular Dynamics and Molecular Mechanics–Generalized Born Surface Area (MM-GBSA) Calculations

Schrödinger Desmond [18] package was used for molecular dynamics simulations using “OPLS4” forcefield as described in past research. The MM-GBSA technique was used to compute the binding free energy of the protein-ligand complexes studied, which integrated molecular mechanics (MM) force fields with a generalized born and surface area continuum solvation solvent model using Schrodinger Prime package [23,33,34,35,36].

### 4.4. Pharmacophore Studies

The pharmacophore model was carried out using Discovery Studio 4.0 software. The protocol of receptor-ligand pharmacophore generation was applied. This protocol used the co-crystallized ligand of MMP-9 (PDB ID: 1GKC) as a reference molecule. The tested compounds were used as a training set. In this protocol, we used the following features in pharmacophore generation: (i) hydrogen bond donor (HBD), (ii) hydrogen bond acceptor (HBA), (iii) hydrophobic aliphatic (HA), (iv) hydrophobic aromatic (HAr), and ring aromatic (RA). Then, the ligand pharmacophore mapping protocol was used in the virtual screening process. The most predictive model was used as 3D queries to identify compounds with high fit values [36,37,38,39].

## Figures and Tables

**Table 1:** The calculated ∆G (binding free energies) of the tested drugs against MMP-2 (PDB ID 1HOV).

	Compound	∆G (kcal.mol^−1^)		Compound	∆G (kcal.mol^−1^)
1	Acetazolamide	−11.39	17	Ganciclovir	−15.93
2	Acyclovir	−15.51	18	Indomethacin	−21.81
3	Ampicillin	−19.31	19	Ketorolac	−18.35
4	Atenolol	−28.20	20	Levocabastine	−22.00
5	Atropine	−15.61	21	Lincomycin	−29.06
6	Aztreonam	−19.01	22	Lornoxicam	−18.51
7	Betaxolol	−26.50	23	Methazolamide	−12.86
8	Brinzolamide	−20.96	24	Methotrexate	−26.00
9	Bromfenac	−18.04	25	Nadolole	−25.86
10	Carteolol	−24.09	26	Pilocarpine	−17.28
11	Cephalexine	−21.07	27	Pindolol	−21.05
12	Ciprofloxacin	−27.87	28	Prednisolone	−19.66
13	Dexamethasone	−20.70	29	Propranolol	−23.93
14	Diclofenac	−17.33	30	Quinidine	−20.97
15	Dorzolamide	−19.06	31	Tizanidine	−15.93
16	Fluconazole	−17.47	32	Voriconazolee	−18.24

**Table 2:** The calculated ∆G (binding free energies) of the tested drugs against the catalytic domain of MMP-9 (PDB ID: 1GKC).

Serial	Compound	∆G (kcal.mol^−1^)	Serial	Compound	∆G (kcal.mol^−1^)
1	Acetazolamide	−15.34	17	Ganciclovir	−28.89
2	Acyclovir	−20.59	18	Indomethacin	−26.12
3	Ampicillin	−30.81	19	Ketorolac	−23.59
4	Atenolol	−27.60	20	Levocabastine	−24.62
5	Atropine	−26.99	21	Lincomycin	−26.74
6	Aztreonam	−29.97	22	Lornoxicam	−21.02
7	Betaxolol	−25.97	23	Methazolamide	−15.99
8	Brinzolamide	−26.07	24	Methotrexate	−27.70
9	Bromfenac	−22.24	25	Nadolole	−25.62
10	Carteolol	−25.96	26	Pilocarpine	−23.69
11	Cephalexine	−24.47	27	Pindolol	−23.56
12	Ciprofloxacin	−25.13	28	Prednisolone	−29.51
13	Dexamethasone	−23.65	29	Propranolol	−24.36
14	Diclofenac	−20.26	30	Quinidine	−28.53
15	Dorzolamide	−20.12	31	Tizanidine	−18.29
16	Fluconazole	−22.03	32	Voriconazolee	−23.46

**Table 3:** The MM-GBSA binding free energies (Kcal.mol^−1^) of MMP-2/atenolol and MMP-9/ampicillin complexes.

	MMP-2/Atenolol		MMP-9/Ampicillin	
	Start	End	Start	End
dG Binding	−38.07	−47.75	−9.35	−26.96
dG binding Coulomb	−22.89	−51.36	39.63	11.89
dG Binding (NS)	−49.77	−52.57	−19.88	−28.70
dG binding (NS) Coulomb	−22.14	−52.42	39.35	13.00

**Table 4:** Fit value of the top four compounds and the co-crystallized ligand of MMP-9 (PDB ID: 1GKC).

Compound	Mapped Features	Fit Value
**NFH**	HBD1, HBD2, HBA1, HBA2, H1, H2	4.74
Ampicillin	HBD1, HBD2, HBA1, HBA2, H1, H2	3.76
Aztreonam	HBD1, HBD2, HBA1, HBA2, H1, H2	4.13
Cephalexine	HBD1, HBD2, HBA1, HBA2, H1, H2	4.06
Lincomycin	HBD1, HBD2, HBA1, HBA2, H1, H2	3.76

## Data Availability

Supplementary Materials are provided.

## Supplementary Materials

The following supporting information can be downloaded at: https://www.mdpi.com/article/10.3390/molecules27113584/s1, Figure S1. Atenolol properties throughout 50 ns simulation in complex with MMP-2; Figure S2. Ampicillin properties throughout 50 ns simulation in complex with MMP-9; Figure S3. RMSF of atenolol throughout 50 ns simulation in complex with MMP-2; Figure S4. RMSF of ampicillin throughout 50 ns simulation in complex with MMP-9; Table S1. Fit values of the full library on MMP-9 pharmacophore.
