# Supplementary material for: Screening a Panel of Topical Ophthalmic Medications against MMP-2 and MMP-9 to Investigate Their Potential in Keratoconus Management
Source: Molecules. 2022 Jun 2;27(11):3584. doi: 10.3390/molecules27113584 (PMC9182209; doi:10.3390/molecules27113584)
Supplement: Supplementary file 1 [file molecules-27-03584-s001.zip › molecules-1711974-supplementary.pdf]

Screening a Panel of Topical Ophthalmic Medications against MMP-2 and MMP-9 to Investigate Their Potential in Keratoconus Management

Amany Belal [1,*], Mohamed A. Elanany [2], Eman Y. Santali [1], Ahmed A. Al-Karmalawy [3], Moustafa O. Aboelez [4], Ali H. Amin [5,6], Magda H. Abdellattif [7], Ahmed B. M. Mehany [8] and Hazem Elkady [9]

[1] Department of Pharmaceutical Chemistry, College of Pharmacy, Taif University, P.O. Box 11099, Taif 21944, Saudi Arabia; eysantali@tu.edu.sa
[2] School of Pharmacy and Pharmaceutical Industries, Badr University in Cairo (BUC), Cairo 11884, Egypt; mohamed.a.elanany@hotmail.com
[3] Department of Pharmaceutical Medicinal Chemistry, Faculty of Pharmacy, Horus University-Egypt, New Damietta 34518, Egypt; akarmalawy@horus.edu.eg
[4] Department of Pharmaceutical Chemistry, Faculty of Pharmacy, Sohag University, Sohag 82524, Egypt; moustafaaboelez@pharm.sohag.edu.eg
[5] Deanship of Scientific Research, Umm Al-Qura University, Makkah 21955, Saudi Arabia; ahamin@uqu.edu.sa
[6] Zoology Department, Faculty of Science, Mansoura University, Mansoura 35516, Egypt
[7] Department of Chemistry, College of Sciences, Taif University, P.O. Box 11099, Taif 21944, Saudi Arabia; m.hasan@tu.edu.sa
[8] Zoology Department, Faculty of Science (Boys), Al-Azhar University, Cairo 11884, Egypt; abelal_81@azhar.edu.eg
[9] Pharmaceutical Medicinal Chemistry & Drug Design Department, Faculty of Pharmacy (Boys), Al-Azhar University, Cairo 11884, Egypt; hazemelkady@azhar.edu.eg
* Correspondence: a.belal@tu.edu.sa or abilalmoh1@yahoo.com or amany.mehani@pharm.bsu.edu.eg

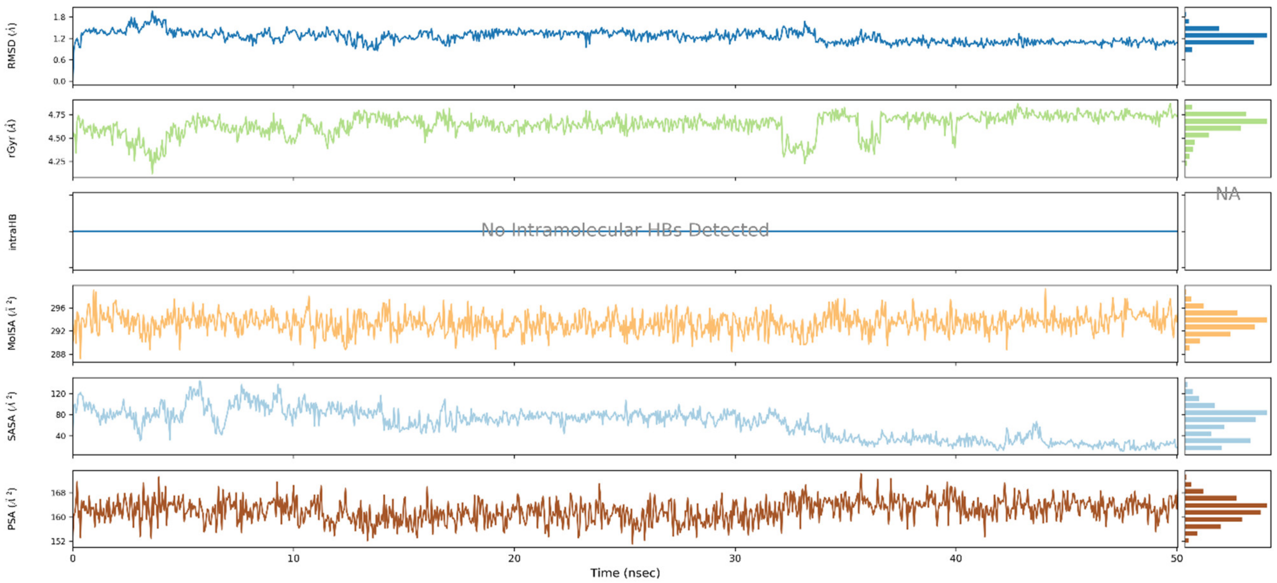

Figure S1. Atenolol properties throughout 50 ns simulation in complex with MMP-2.

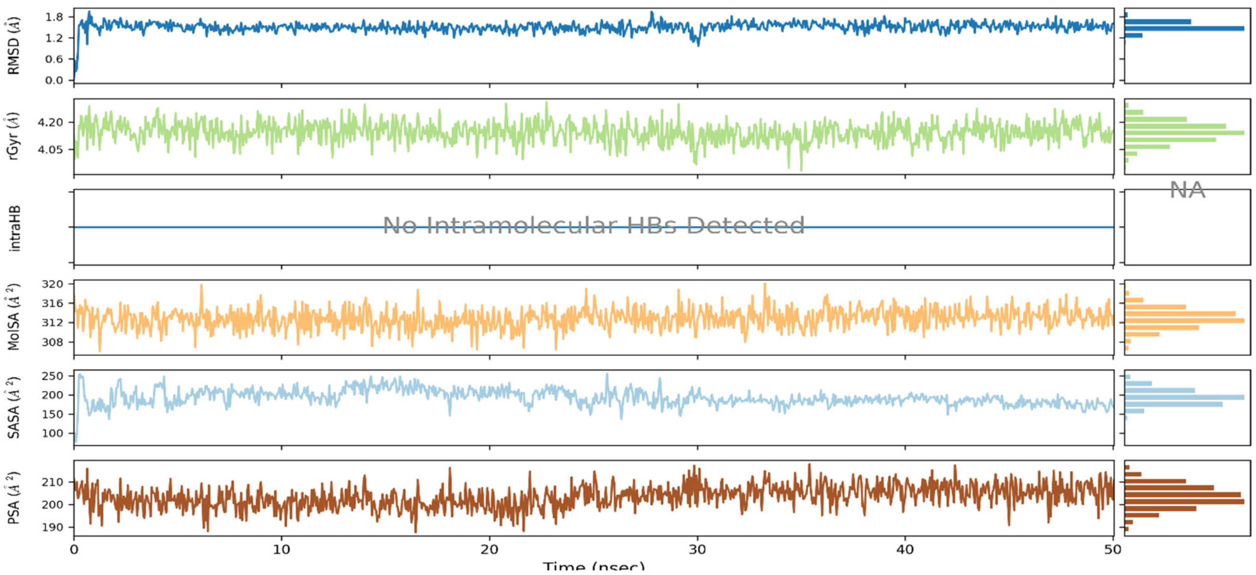

Figure S2. Ampicillin properties throughout 50 ns simulation in complex with MMP-9.

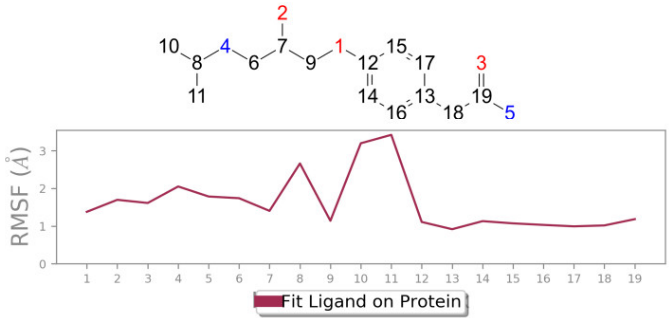

Figure S3. RMSF of atenolol throughout 50 ns simulation in complex with MMP-2.

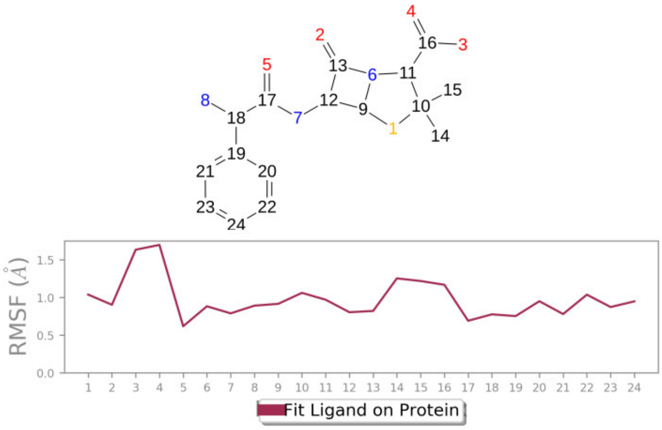

Figure S4. RMSF of ampicillin throughout 50 ns simulation in complex with MMP-9.

Table S1. Fit values of the full library on MMP-9 pharmacophore.

Serial	Compound	Fit value	Serial	Compound	Fit value
1	Acetazolamide	2.67	17	Ganciclovir	2.65
2	Acyclovir	2.62	18	Indomethacin	2.82
3	Ampicillin	3.76	19	Ketorolac	2.61
4	Atenolol	3.22	20	Levocabastine	2.60
5	Atropine	3.41	21	Lincomycin	3.76
6	Aztreonam	4.13	22	Lornoxicam	3.14
7	Betaxolol	3	23	Methazolamide	2.97
8	Brinzolamide	2.78	24	Methotrexate	3.66
9	Bromfenac	3.08	25	Nadolol	3.69
10	Carteolol	2.96	26	Pilocarpine	2.92
11	Cephalexine	4.06	27	Pindolol	2.94
12	Ciprofloxacin	2.59	28	Prednisolone	3.53
13	Dexamethasone	3.63	29	Propranolol	2.95
14	Diclofenac	2.90	30	Quinidine	3.06
15	Dorzolamide	2.79	31	Tizanidine	2.84
16	Fluconazole	2.54	32	Voriconazole	3.45
